# Adverse changes in left ventricular structure begin at normotensive systolic blood pressures: a high resolution MRI study

**DOI:** 10.1186/1532-429X-17-S1-M11

**Published:** 2015-02-03

**Authors:** Antonio de Marvao, Timothy J Dawes, Wenzhe Shi, Giuliana Durighel, Daniel Rueckert, Stuart A Cook, Declan P O'Regan

**Affiliations:** 1 Medical Research Council Clinical Sciences Centre, Imperial College London, London, UK; 2 Department of Computing, Imperial College London, London, UK; 3 Duke-NUS Graduate Medical School, Singapore, Singapore

## Background

The prevalence of hypertension has doubled over the previous decade and represents a significant public health problem worldwide. Excess cardiovascular risk due to increased blood pressure shows no evidence of a threshold to at least 115/75 mmHg. Left ventricular (LV) hypertrophy has a strong association with essential hypertension and is an independent risk factor for mortality. However, there is little data on whether systolic blood pressure (SBP) influences LV morphology and function in a healthy population. Furthermore it is not clear if any phenotypical changes are part of an adaptive response to increasing afterload or the beginning of a maladaptive process that ultimately leads to cardiomyopathy.

## Methods

A total of 1258 volunteers (54% females, mean age 40.6±12.8 years) without self-reported cardiac disease underwent high-spatial resolution 3-dimensional cardiac magnetic resonance imaging with computational modeling to characterise LV structure and function. Voxelwise relative wall thickness (RWT), fractional wall thickening (FWT), longitudinal function, and LV shape were the dependent variables. Changes in these parameters associated with SBP were analysed using multivariable linear regression models with adjustment for body surface area, gender, race, age and multiple testing. Results are presented as standardized β coefficients in the anatomic regions where the corrected p-value was <0.05.

## Results

**Figure 1:**
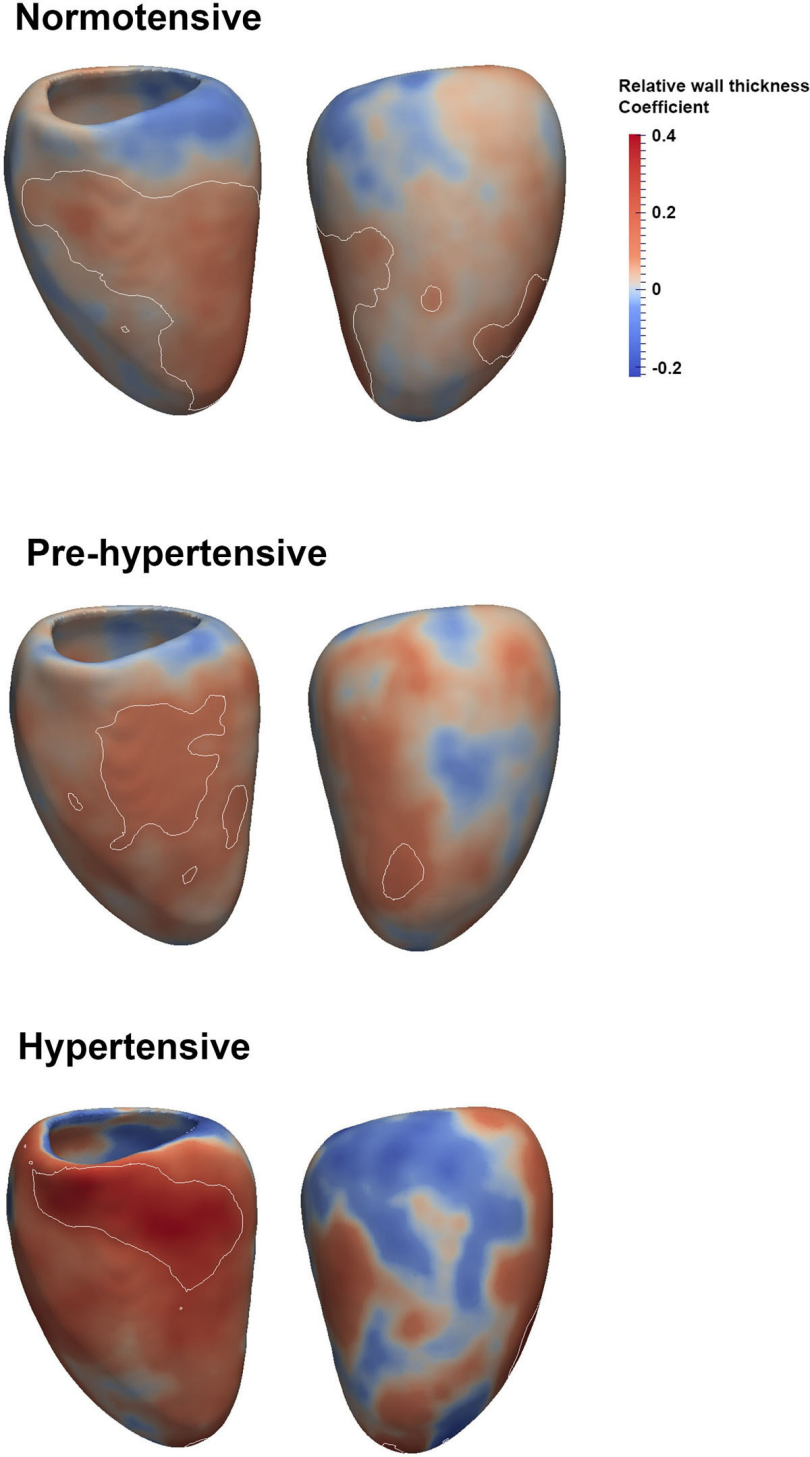
Correlation coefficients between relative wall thickness and SBP have been mapped onto the surface of the left ventricle in normotensive, pre-hypertensive and hypertensive sub-groups. In each pair the left image shows the septal wall and the right image the lateral wall. A progressive concentric hypertrophic response is present within the septum which increases in magnitude in each category of SBP. The white isoline represents the areas within which statistical significance was reached (p-value <0.05).

In our cohort 8.3% of subjects had stage 1 or 2 systolic hypertension (≥140 mmHg), 38.4% had pre-hypertension (120 - 139 mmHg) and 53.3% were normotensive (<120 mmHg). SBP was independently associated with an asymmetric increase in RWT in the three sub-groups (Figure 1). In normotensive subjects concentric hypertrophy occurred across a large area of the septum and lateral wall (β = 0.07). In pre-hypertensives the mid-ventricular septum had the most significant increase in RWT (β = 0.10), while in hypertensives this also occurred in the basal septum (β = 0.31). Males had a greater hypertrophic response than females (β = 0.67). Morphological changes in normotensive subjects occurred in the same anatomic regions where decreased radial and longitudinal function was observed in the pre-hypertensive and hypertensive subjects.

**Figure 2:**
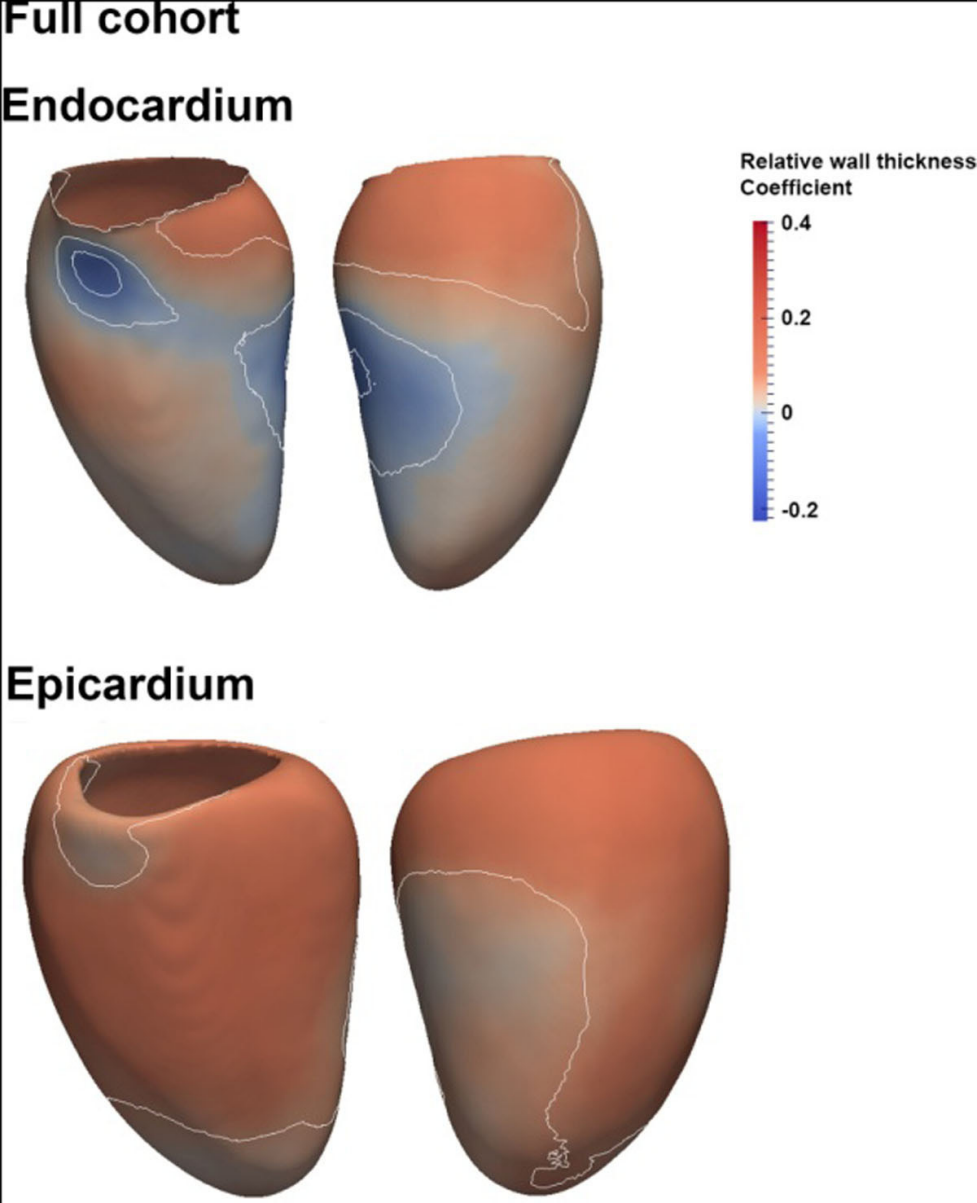
Correlation coefficients between endocardial volume (upper row) and epicardial volume (lower row) vs SBP are shown for the whole study cohort. The endocardial surface dilates in response to rising SBP except for an area that extends from the basal septum to the mid-ventricular anterior wall where hypertrophy occurs at the expense of the cavity. The epicardial surface also shows a global expansion in association with SBP but which is less marked in the lateral wall. These changes are consistent with a concentric hypertrophic response throughout the septum and anterior wall. The white isoline represents the areas within which statistical significance was reached (p-value <0.05)

## Conclusions

In a healthy population geometric changes in the LV associated with SBP may not be purely homeostatic and share morphological features of the hypertensive phenotype. Asymmetric concentric hypertrophy was detected even in normotensives although function was preserved. Early adverse myocardial adaptations to SBP may contribute to the excess risk of disease recognised even in apparently healthy populations.

## Funding

The study was supported by the Medical Research Council, UK, the National Institute for Health Research (NIHR) Biomedical Research Centre based at Imperial College Healthcare NHS Trust and Imperial College London, UK, a British Heart Foundation, UK, project grant (PG/12/27/29489) and special grant (SP/10/10/28431), and a Wellcome Trust-GSK Fellowship Grant.

